# Neoadjuvant and Adjuvant Immunotherapy: Opening New Horizons for Patients With Early-Stage Non-small Cell Lung Cancer

**DOI:** 10.3389/fonc.2020.575472

**Published:** 2020-10-09

**Authors:** Rilan Bai, Lingyu Li, Xiao Chen, Naifei Chen, Wei Song, Jiuwei Cui

**Affiliations:** Cancer Center, The First Hospital of Jilin University, Changchun, China

**Keywords:** non-small cell lung cancer, neoadjuvant therapy, response evaluation, immune checkpoint inhibitor, predictive biomarker

## Abstract

Lung cancer is the most common malignant tumor with the highest mortality, and about 84% are non-small cell lung cancer (NSCLC). However, only a small proportion of patients with newly diagnosed lung tumors can receive curative surgery and have a high risk of postoperative recurrence. At present, there are many perioperative treatment methods being continuously explored, such as chemotherapy and targeted therapy, continuously enriching the content of neoadjuvant and adjuvant therapy in early-stage NSCLC. But disappointingly, for patients with driver gene mutation, the significant disease-free survival (DFS) benefit of targeted drugs failed to translate into overall survival (OS) benefit, and for negative patients, chemotherapy has reached a plateau in improving efficacy and survival. Immunotherapy represented by immune checkpoint inhibitors (ICIs) has been researched in more and more clinical trials in patients with early-stage operable disease, gradually enriching the existing treatments. This review focuses on the research progress of clinical trials of neoadjuvant and adjuvant therapy with ICIs in early-stage NSCLC, the exploration of response evaluation and predictive biomarkers, and the urgent problems to be solved in the future.

## Introduction

According to global cancer statistics in 2018, lung cancer is the most common (11.6% of all cases) malignant tumor with the highest mortality (18.4% of total cancer deaths) (1), of which about 84% is non-small cell lung cancer (NSCLC). However, only about 20–30% of newly diagnosed lung tumors can receive radical surgery, and many of them have a high risk of recurrence (25–70%) due to the presence of preoperative micrometastases (2). At present, there are many perioperative treatment methods being continuously explored to reduce the risk of recurrence and improve long-term survival, such as targeted therapy and chemotherapy, which are continuously enriching the content of neoadjuvant and adjuvant therapy in early-stage NSCLC, making the treatment lines continuously advanced and bringing a brand-new different era for operable patients. For patients with driver gene-positive early-stage lung cancer, a number of studies have been tried and made breakthrough results. The phase III ADJUVANT study (3), phase II EVAN study (4), and EMERGING study (CTONG 1103) (5) all achieved positive results for disease-free survival (DFS) or progression-free-survival (PFS) in perioperative treatment with targeted drugs, suggesting that targeted therapy can change the treatment pattern of early-stage lung cancer. However, the latest long-term follow-up results showed that significant DFS benefit failed to translate into overall survival (OS) benefit. For patients with negative driver gene, the National Comprehensive Cancer Network (NCCN) guidelines recommend conventional adjuvant chemotherapy for patients with postoperative pathological stage IIB or higher and adjuvant chemotherapy for high-risk patients with stage IB/IIA (6). Several meta-analyses suggested that the survival benefit of neoadjuvant chemotherapy is comparable to that of postoperative adjuvant chemotherapy, with the 5-year survival rate increased by about 4–8% (7, 8). It can be seen that the benefit is unsatisfactory, and despite surgery and adjuvant therapy, about 20–30% of patients with stage I, 50% with stage II, and 60% with stage IIIA die within 5 years (9), which led researchers to focus on exploring new drugs of neoadjuvant and adjuvant therapies. Immunotherapy represented by immune checkpoint inhibitors (ICIs) has been researched in more and more clinical trials in patients with early-stage operable disease, gradually enriching the existing treatments, and these trials found that it has more advantages in killing tumor, preventing postoperative recurrence, and improving survival. This review focuses on the research progress of clinical trials of neoadjuvant and adjuvant therapy with ICIs in early-stage NSCLC, the exploration of response evaluation and predictive biomarkers, and the urgent problems to be solved in the future.

## Advantages and Disadvantages of Neoadjuvant Immunotherapy

The goals of neoadjuvant therapy include decreasing tumor TNM stage, increasing R0 resection rate, controlling micrometastases, improving DFS and overall OS, and assessing drug efficacy or conducting drug susceptibility studies. Recent studies have found that the immunosuppressive tumor microenvironment (TME) already exists in tumor tissues of stage I NSCLC. The immune cell composition and phenotype in the early TME have changed significantly, including T cells, natural killer (NK) cells, and tumor-infiltrating myeloid cells (TIM). The researchers showed that the lesions are enriched in a variety of inhibitory cells, such as programmed cell death-ligand 1 (PD-L1) ^*hi*^CD64^*hi*^CD14^*hi*^PPARγ^*hi*^ IL-6^*hi*^ macrophages, CD1c^+^DC, CD39^*hi*^CD38^*hi*^PD1^*hi*^CTLA^*hi*^ T regular cell (Treg), and exhausted T cells, and depleted of cells that can effectively exert anti-tumor effector functions, such as CD141^+^ dendritic cell (DC), CD16^+^ monocytes, NK cells, and granzyme B^+^ effector cells. These differences may synergistically promote the immunosuppressive microenvironment. Therefore, immunotherapy is essential for patients with early-stage tumor. Neoadjuvant therapy with ICIs given before surgical resection of early-stage NSCLC can induce a more sustained anti-tumor T cell immune response, thereby more effectively preventing tumor recurrence (10). (i) Neoadjuvant immunotherapy can increase the number of activated tumor-specific CD8^+^ T cells, which can release more new tumor antigens while killing tumors, and then these antigens are presented to specific effector T cells of tumors at different sites (primary tumor, metastases, circulation); (ii) activated T cells can reach micrometastases through blood vessels and lymphatic vessels, triggering a range of specific anti-tumor immune responses; (iii) in addition, compared with postoperative adjuvant therapy, the structure of the lymphatic system around the lung cancer before resection is relatively intact, providing a greater chance of interaction between tumor cells and immune cells. Moreover, the presence of a wider repertoire of tumor neoantigens can enhance immune recognition and produce a strong anti-tumor immune response and early immune memory. Preclinical studies and early clinical trials seem to support the neoadjuvant approach. Nevertheless, the exploration of immunotherapy in the treatment of early-stage lung cancer also has some risks: delaying surgery and making the disease progress; increasing the difficulty and risk of surgery, such as increased pleural adhesions; and increasing intraoperative and postoperative complications and overtreatment. Therefore, it is necessary to deeply explore the efficacy and safety of neoadjuvant immunotherapy to weigh the benefit/risk ratio to maximize the clinical benefit of the patients.

However, neoadjuvant immunotherapy also has some disadvantages. Firstly, it remains unknown whether it can effectively improve the long-term survival of the patient. Secondly, neoadjuvant immunotherapy may have an impact on the feasibility of surgery, such as delaying surgery or risk of progression before surgical treatment, and may increase the possibility of surgical complications and overtreatment. In addition, there are challenges for optimal response assessment and biomarker exploration of neoadjuvant immunotherapy, which may limit the application and development of neoadjuvant immunotherapy to some extent.

## Review and Perspective on Neoadjuvant Therapy With Immune Checkpoint Inhibitors for Ear*Ly-Stage* Non-Small Cell Lung Cancer

### Neoadjuvant Monotherapy With Immune Checkpoint Inhibitors

The CheckMate 159 study (11) was the first research to prospectively explore the feasibility and safety of neoadjuvant therapy with ICIs in 22 patients with treatment-naive and resectable stage I–IIIA NSCLC, with 20 patients [2 partial response (PR) and 18 stable disease (SD)] undergoing curative surgery after neoadjuvant nivolumab and 45% achieving major pathologic response (MPR). At follow-up, the recurrence rate within 18 months was 73%, the OS rate was 95%, and the 24-month relapse-free survival (RFS) estimated by the Kaplan–Meier curve was 69%. Although the sample size was small, this trial confirmed the safety of neoadjuvant immunotherapy for NSCLC, laying the foundation for subsequent studies (11–13). The phase II LCMC3 study (14) evaluated the safety and efficacy of neoadjuvant atezolizumab in 101 patients with resectable stage IB–IIIA NSCLC with 7% being PR, 89% being SD, 18% being MPR, and 5% being pathologic complete response (pCR), and the therapy was well tolerated by patients with 6% of immune-related adverse event (irAE) of grade ≥3. The phase IB ChiCTR-OIC-17013726 study (15) treated 22 patients with resectable IB–IIIA stage squamous NSCLC with neoadjuvant sintilimab. Postoperative pathological results showed that 45.5% achieved MPR and 18.2% achieved pCR, and the objective response rate (ORR) was 13.6%. Comparison of PET–CT before and after treatment showed that 8 of 9 patients with 30% decrease in tumor metabolism uptake (TMU) achieved MPR, while no MPR was found in 11 patients with less than 30% decrease or increase in TMU, suggesting that changes in TMU on PET–CT before and after treatment may predict postoperative MPR status. As a whole, sintilimab has shown good safety profiles in neoadjuvant therapy for resectable NCSLC. Another study by Li et al. (16) also showed that neoadjuvant sintilimab treatment in NSCLC patients was well tolerated, with an MPR of 40.5% and a pCR of 16.2%. A decrease in TMU on PET–CT rather than a change in the sum of lesion diameters (SLD) was also identified as a predictor of pathological response to anti-programmed cell death-1 (PD-1) therapy in early-stage NSCLC in another study. In addition, it was found that primary lesions and metastatic lymph nodes may have a large heterogeneity in response to neoadjuvant sintilimab treatment. The indications of sintilimab in the treatment of early-stage lung cancer need to be intensively studied in the future, and key factors to overcome heterogeneous responses and delay disease progression need to be explored.

### Immune Checkpoint Inhibitor-Based Neoadjuvant Combination Therapy

Given the limited efficacy of neoadjuvant immune monotherapy and the synergistic effect of chemotherapy and immunotherapy in cancer therapy, several trials have been designed to assess the efficacy and safety of immunotherapy combined with chemotherapy in the neoadjuvant treatment of early-stage NSCLC. A phase II study exploring the use of neoadjuvant atezolizumab in combination with chemotherapy (nab-paclitaxel and carboplatin) in resectable stage IB–IIIA NSCLC, with preliminary results in 14 patients, has reported radiographic PR in 57% of patients and MPR in 7 of 14 patients (50%), including 3 pCR, and is ongoing (17). The phase II NADIM study (18) is the first study to explore the efficacy and safety of nivolumab in combination with paclitaxel and carboplatin in neoadjuvant/adjuvant therapy in patients with resectable stage IIIA NSCLC. After neoadjuvant combination therapy, 93% patients had downstaging, and R0 resection was performed in 41/46 patients; MPR was 83% and pCR reached 71% after operation; PR was 72% and CR was 6.5%; survival data showed that in the ITT population, 12-month PFS was 96%, 18-month PFS rate was 81%, and 18-month OS rate was 91%. In summary, the MPR and survival data of the study reached unprecedented new breakthroughs. The CheckMate 77T study further expanded the sample size on the basis of the NADIM study to demonstrate the exact efficacy of this neoadjuvant modality in the context of a phase III study. In this study, II–IIIA or IIIB (T3N2) epidermal growth factor receptor (EGFR)/anaplastic lymphoma kinase (ALK)-negative NSCLC, the primary study endpoint was event-free survival (EFS) assessed by an independent review. Preliminary results from a small clinical trial (NCT03366766) at the 2020 American Society of Clinical Oncology (ACSO) meeting showed that nivolumab combined with platinum doublet was well tolerated in 13 patients with stage IB–IIIA resectable NSCLC; postoperative MPR appeared in 11/13 patients (85%) and pCR in 5/13 (38%); imaging response rate was 46% (PR 5, CR 1), and no recurrence was observed after 10 months of follow-up.

In addition, neoadjuvant strategies for the combination of dual ICIs are also being explored. The phase II NEOSTAR study (19) assessed the efficacy of neoadjuvant nivolumab (group N) and nivolumab in combination with ipilimumab (group NI) in 44 patients with stage I–IIIA resectable NSCLC. Overall MPR was 24%, overall MPR + pCR was 25% (N vs. NI = 17% vs. 33%), pCR was 18% (N vs. NI = 9% vs. 29%), ORR was 20% (N vs. NI = 22% vs. 19%), and ORR was positively correlated with MPR (*p* < 0.001). In 37 patients with surgical resection, MPR was 30% (N vs. NI = 19% vs. 44%), and the group NI had a significantly lower percentage of viable tumor cells than the group N (20% vs. 70%, *p* = 0.077). Moreover, markers analysis showed that CD3^+^CD103^+^ memory cells (81.2% vs. 54.4%, *p* = 0.021) and the proportion of CD8^+^T cells (56.2% vs. 38.3%, *p* = 0.057) significantly increased in combination immunotherapy. In terms of safety, treatment-related adverse events (TRAEs) of grade 3 to 5 included death from bronchopleural fistula caused by pneumonia associated with steroid therapy (one case, grade 5, group N); grade 3 pneumonia, hypoxia, and hypermagnesemia (each one case, group N); and grade 3 diarrhea (one case, group NI). Thus, neoadjuvant combination therapy seems safe and more effective compared with immune monotherapy. Overall, the NEOSTAR study showed that the complexity of surgery and lung function of patient were not affected by neoadjuvant immunotherapy, and the overall resection rate was comparable to the effect of neoadjuvant chemotherapy, as well as there was no increase in unacceptable toxicity or perioperative morbidity and mortality. However, five patients who failed to undergo surgical resection and one patient who died during perioperative period suggested that neoadjuvant immune monotherapy or combination therapy for patients with resectable NSCLC should be carefully selected after balancing the factors of treatment efficacy, surgical difficulty, and risk.

The corresponding results of completed clinical trials of neoadjuvant therapy with ICIs for resectable NSCLC are detailed in Table 1.

**TABLE 1:** The results of completed clinical trials of neoadjuvant therapy with ICIs for resectable NSCLC.

**Clinical trial**	**Phase**	**Stage**	**Intervention used**	**Sample size**	**Primary endpoint**	**Primary outcomes**
CheckMate 159	I	I–IIIA	Nivolumab	22	Safety and feasibility	MPR: 45%, pCR: 10%
LCMC3	II	IB–IIIA	Atezolizumab	101	MPR	MPR: 18%, pCR: 5%
Li et al. (13)	II	IA–IIIB	Sintilimab	40	Safety	MPR: 40.5%, pCR: 16.2%
Li et al. ChiCTR-OIC-17013726	IB	IA–IIIA	Sintilimab	22	Drug-related adverse event; surgery complications; no-delay surgery rate	MPR: 45.5%, pCR: 18.2%
NADIM	II	IIIA	Nivolumab + chemotherapy	46	PFS at 24 months	MPR: 83%, pCR: 71%
NEOSTAR	II	I–IIIA	Nivolumab vs. nivolumab + ipilimumab	44	MPR	MPR: 24%, pCR: 18%

*NSCLC, non-small cell lung cancer; MPR, major pathologic response; pCR, pathologic complete response; ICIs, immune checkpoint inhibitors; PFS, progression-free survival.*

### The Safety and Efficacy Analysis of Neoadjuvant Therapy With Immune Checkpoint Inhibitors in Early-Stage Non-small Cell Lung Cancer

Although neoadjuvant immunotherapy has attracted much attention in the surgical treatment of tumors, it still deserves attention for the possible technical challenges during surgery and drug side effects during or after treatment (such as pneumonia and endocrinopathy). To solve the problems, the surgical conditions, perioperative safety, and complications after neoadjuvant immunotherapy were comprehensively analyzed in multiple studies. In the NA00092076 study, the proportion of thoracotomy was 70% (14/20), and the incidence of postoperative complications was 50%, of which the most common was atrial arrhythmia (30%), but no surgery-related death occurred (20). In the NEOSTAR study, thoracotomy accounted for 73% (27/37), and the combination therapy significantly reduced the probability of subsequent surgical treatment (two cases in group N, five cases in group NI). The incidence of postoperative complications was 21.6%, of which the most common was persistent air leak, with a surgery-related mortality rate of 3%. A prospective study by Yang et al. (21) showed that compared with induction with standard neoadjuvant chemotherapy, the morbidity and mortality were not increased in 13 patients with stage II–IIIA NSCLC who received neoadjuvant treatment with ipilimumab. A recent study confirmed that neoadjuvant immunotherapy of nivolumab in NSCLC patients did not increase the difficulty of surgery, blood loss was usually low, and there was no unexpected morbidity with atrial fibrillation in six patients (30%); postoperative pneumonia, empyema, and persistent air leak in one patient each (5% each); and low incidence of serious irAEs. However, it should be noted that 54% of the cases were converted to thoracotomy from robot-assisted thoracic surgery (RATS) or video-assisted thoracic surgery (VATS), mostly due to pleural adhesions, perihilar inflammation, and fibrosis (20). For the immunotherapy cycles, the CheckMate 159 study, NEOSTAR study, and NADIM study administered 1–2, 3, and 3 cycles before operation, respectively. The final results did not affect the timing of operation, and relevant studies are still being explored.

The current studies of neoadjuvant therapy with ICIs in NSCLC are all phase I/II exploratory clinical studies, most of which are single-arm designs with a small sample size (10 to 101 cases). Preliminary results showed that the safety of immunotherapy was good, but the MPR was low. Although the MPR of neoadjuvant therapy with nivolumab reached 45% in the CheckMate 159 study, the subsequent LCMC3 study and NEOSTAR study failed to replicate the results. The NADIM study of immunotherapy combined with chemotherapy achieved the highest MPR and pCR so far and all performed surgery as scheduled. But the sample size of this study was small and the incidence of specific adverse events was not published. The MPR of dual immunotherapy regimen could improve to some extent, but it significantly reduced the chance of patients receiving subsequent surgical treatment, and the CheckMate 617 study of dual immunotherapy was terminated early. Therefore, the selection of immunotherapy regimen needs to be carefully selected after balancing factors of treatment efficacy, safety, and surgery rate. Overall, RECIST criteria and MPR assessment showed good anti-tumor activity and safety of neoadjuvant therapy with ICIs, which prompted an important step toward longer-term survival in early-stage NSCLC although the reliability and reproducibility of the results have yet to be further confirmed.

### Ongoing Trials of Neoadjuvant Therapy With Immune Checkpoint Inhibitors in Patients With Resectable Non-small Cell Lung Cancer

Given the current breakthrough, multiple studies of neoadjuvant therapy with ICIs for stage II/III NSCLC are planned or ongoing, which will provide more data on safety and efficacy and contribute to the development of more effective treatment strategies. The primary endpoints of most studies are MPR, EFS, or DFS, while a few studies set to OS. The trials explored different neoadjuvant immunotherapy regimens. For example, the MK3475-223 (NCT02938624), TOP 1501 (NCT02818920), IONESCO (NCT03030131), Columbia University (NCT02716038), PRICNEPS (NCT0299457), and a phase II study (NCT02927301) are studying treatment using ICIs alone, the last study of which explored drug efficacy and preliminary results of 54 patients showed that the MPR rate was 20% and the tolerability was good with only one patient having delayed surgery due to pneumonia. Six trials are exploring the efficacy and safety of ICIs combined with chemotherapy in early-stage NSCLC, including phase II SAKK 16/14 trial (NCT02572843) and NADIM-II clinical trial (NCT03838159), phase III IMpower030 study (NCT03456063), CheckMate 816 study (NCT02998528), Keynote-671 study (NCT03425643), and AEGEAN study (NCT03800134). There are some other treatment options. Preliminary recent results of SAKK 16/14 showed an ORR of 44.8% in the chemotherapy stage compared with 59.7% in the immune neoadjuvant stage; 81% of patients underwent surgery (the most important reason for not undergoing surgery was disease progression, accounting for 33.3%). Encouragingly, the study showed that the 1-year EFS rate was 73.3%, surpassing the previous rate of about 50% in patients with stage IIIA disease. Thus, the treatment mode of chemotherapy with sequential immunotherapy is worthy of further expanding the sample size to demonstrate its exact benefit and looks forward to the publication of subsequent results. In addition to neoadjuvant chemotherapy combined with immunotherapy, the efficacy and safety of other regimens have also been explored. The phase II randomized study NeoCOAST is underway to compare the clinical activity and feasibility of durvalumab ± oleclumab (MEDI9447) or monalizumab (IPH2201) or danvatirsen in patients with resectable stage I–IIIA NSCLC. Unfortunately, a third arm of the CheckMate 816 study in patients receiving nivolumab plus ipilimumab has been discontinued due to intolerance in patients. Besides, radiotherapy can enhance the therapeutic effect of local lesions, reduce micrometastatic lesions, increase the immunogenicity of tumors, and also may lead to the upregulation of PD-L1 expression in tumors. Therefore, several trials (NCT03110978, NCT03237377, and NCT02904954) are evaluating the synergistic anti-tumor effect of radiotherapy with ICIs in early-stage NSCLC.

In addition to the different treatment regimens, several studies with neoadjuvant therapy continue ICI therapy in the adjuvant setting for 1 year, such as the SAKK 16/14 trial, IMpower030, Keynote-671, and NADIM-II, or perform consolidation therapy with ICI after adjuvant therapy, such as the TOP 1501 trial. But they may affect the evaluation of the efficacy of neoadjuvant therapy, needing more well-designed studies to confirm the results. The details of ongoing clinical trials of neoadjuvant ICIs and ICI-based combination therapy for earlier-stage NSCLC are listed in Table 2.

**TABLE 2:** Ongoing clinical trials of neoadjuvant therapy with ICIs for earlier-stage NSCLC.

**Clinical trial**	**Phase**	**Stage**	**Intervention used**	**Estimated sample size**	**Primary endpoints**
MK3475-223 (NCT02938624)	I	I–II	Pembrolizumab	28	Toxicity, MPR
TOP 1501 (NCT02818920)	II	IB–IIIA	Pembrolizumab	32	Surgical feasibility
IONESCO (NCT03030131)	II	IB-II	Durvalumab	81	R0 resection
Columbia University (NCT02716038)	II	IB–IIIA	Atezolizumab	30	MPR
PRICNEPS (NCT0299457)	II	IB–IIIA	Atezolizumab	60	Toxicity
NCT02927301	II	IB–IIIA	Atezolizumab	180	MPR
NeoCOAST	II	I–IIIA	Durvalumab ± oleclumab (MEDI9447) or monalizumab (IPH2201) or danvatirsen	160	–
SAKK 16/14 (NCT02572843)	II	IIIA (N2)	Durvalumab + chemotherapy	68	EFS
NADIM-II (NCT03838159)	II	IIIA/IIIB with T3N2	Chemotherapy + nivolumab vs. chemotherapy	90	pCR
IMPower030 (NCT03456063)	III	II–IIIB	Chemotherapy + atezolizumab vs. chemotherapy + placebo	374	MPR, EFS
CheckMate 816 (NCT02998528)	III	IB–IIIA	Chemotherapy vs. chemotherapy + nivolumab vs. nivolumab + ipilimumab	350	EFS, pCR
Keynote-671 (NCT03425643)	III	II–IIIB	Chemotherapy + pembrolizumab vs. chemotherapy	786	EFS, OS
AEGEAN (NCT03800134)	III	IIA–IIIB	Chemotherapy + durvalumab vs. chemotherapy + placebo	300	MPR
NCT03110978	I/II	I–IIA	Radiotherapy + nivolumab vs. radiotherapy	140	EFS, secondary malignancy, and death
NCT03237377	II	IIIA	Durvalumab + radiation or durvalumab + tremelimumab + radiation	32	Safety, feasibility
NCT02904954	III	II–III	Durvalumab with or without radiotherapy	-	DFS

*NSCLC, non-small cell lung cancer; MPR, major pathologic response; pCR, pathologic complete response; ICIs, immune checkpoint inhibitors; EFS, event-free survival; OS, overall survival; DFS, disease-free survival.*

## The Research Progress of Adjuvant Therapy With Immune Checkpoint Inhibitors in Patients With Resectable Non-Small Cell Lung Cancer

At present, the study of adjuvant therapy for NSCLC is in the exploratory stage with no mature research result. There are two main ongoing trials of adjuvant therapy with anti-PD-1 agents, ANVIL and PEARLS, and three main trials of that with anti-PD-L1 agents (22–24) for earlier-stage NSCLC, all of which are ICIs with or without chemotherapy. The primary endpoint of most studies is DFS. The specific details of these trials are exhaustively described in Table 3. All clinical trials of adjuvant immunotherapy are expected to be completed during 2024–2027; thus, the role of anti-PD-1/PD-L1 drugs in adjuvant therapy may remain unclear over a period of time. In addition, there are no ongoing clinical trials that compare the efficacy and safety data of neoadjuvant immunotherapy against the adjuvant immunotherapy strategies, and some trials have followed adjuvant therapy after neoadjuvant therapy, all of which still limit the judgment of the effectiveness of the treatments to some extent. Adjuvant therapy, neoadjuvant therapy, or the combination of both, which is the best treatment strategy, remains unknown. The results of these studies may have a substantial impact on the clinical practice of patients with locally resected NSCLC, and the development of more large-scale prospective clinical trials is expected in the future.

**TABLE 3:** Ongoing clinical trials of adjuvant therapy with ICIs for earlier-stage NSCLC.

**Clinical trial**	**Phase**	**Stage**	**Intervention used**	**Estimated sample size**	**Primary endpoints**
ANVIL (NCT02595944)	III	IB–IIIA	Nivolumab vs. observation with or without adjuvant chemotherapy	903	OS, DFS
PEARLS/Keynote-091 (NCT02504372)	III	IB–IIIA	Pembrolizumab vs. placebo with or without adjuvant chemotherapy	1,380	DFS
IMpower010 (NCT02486718)	III	IB–IIIA	Atezolizumab + chemotherapy vs. best supportive care + chemotherapy	1,280	DFS
BR31 (NCT02273375)	III	IB–IIIB	Durvalumab vs. placebo with or without adjuvant chemotherapy	1,100	DFS in PD-L1-positive patients and in all randomized patients

*NSCLC, non-small cell lung cancer; ICIs, immune checkpoint inhibitors; OS, overall survival; DFS, disease-free survival; PD-L1, programmed cell death-ligand 1.*

## Response Evaluation to Neoadjuvant Immunotherapy

Neoadjuvant clinical trial endpoints include pCR, DFS, and OS. Pathological response is considered to improve the efficiency of the study and predict survival, which is approved by the United States Food and Drug Administration (FDA) and European Medicines Agency for survival surrogate endpoints in neoadjuvant breast cancer studies. However, the pCR of neoadjuvant chemotherapy for lung cancer in 15 studies is only 4%, which greatly limits its application (25). In addition, OS is considered the most widely accepted study endpoint and the “gold standard” for demonstrating the clinical benefit of any cancer treatment; DFS, a composite endpoint combining time to disease recurrence and OS, is commonly used as a surrogate for OS. However, the use of these endpoints to predict clinical benefit in early-stage NSCLC is problematic and may slow down the process of drug development. First, in the neoadjuvant setting for localized NSCLC, it is difficult to include a large number of patients to have sufficient power to identify a difference in survival. Second, it may take many years to reliably establish improvements in OS and DFS. Therefore, identifying surrogate endpoints that do not require long-term follow-up and can accurately predict OS is important. Recently, researchers have proposed MPR, which refers to neoadjuvant therapy-induced tumor response with pathological residual tumor less than 10%, and have verified its effectiveness in neoadjuvant chemotherapy. Residual tumor cells were positively associated with the risk of death after neoadjuvant chemotherapy, but not with the risk of death from surgery alone. A follow-up report also showed that MPR was associated with OS (residual tumor cells >10%, HR 2.39, *p* = 0.05). The study of neoadjuvant chemotherapy for NSCLC conducted by Pataer et al. (26) also identified that the OS rate and DFS of patients in the MPR group were significantly greater than those in the non-MPR group, and MPR was still associated with survival when controlling for pathological stage. Therefore, MPR is considered a surrogate endpoint measure for studies of neoadjuvant therapy in patients with NSCLC, and multiple studies of neoadjuvant immunotherapy have selected MPR as a primary or secondary study endpoint (11, 27). In the NEOSTAR study, RECIST-assessed disease response was positively correlated with MPR (*p* < 0.001); the results of the CheckMate 159 study at 34.6 months of follow-up showed that neoadjuvant MPR with nivolumab was associated with recurrence rate. However, the association between MPR response and DFS or OS is not clear, still needing longer follow-up verification; and when comparing different clinical trials, how to accurately measure and evaluate risk based on different HR values is not uniform.

Besides, in the clinical efficacy evaluation, considering their peculiar mechanisms of action different from chemotherapy, immunotherapies make the inconsistency between pathological and imaging assessment and would develop atypical response patterns that extend beyond those of cytotoxic agents, such as pseudoprogression (PsPD), delayed responses, etc., which makes it difficult to accurately grasp the response rate of immunotherapy by traditional imaging assessment alone. Although the situation may be improved with the development of techniques such as PET–CT, many difficulties are still faced. A study of neoadjuvant nivolumab in NSCLC showed that ORR by radiographic assessment at surgery was only 10% (2/20), while MPR by pathological assessment reached 45% (9/20) (11). Therefore, precise evaluation of neoadjuvant immunotherapy response is particularly important for surgical treatment. Attempts have been made to develop a new quantitative immune-related pathological response criterion (irPRC) to standardize the assessment of pathological response after neoadjuvant treatment with ICIs for NSCLC. This standard added the area of the regression lesions to the areas of residual active tumor and necrosis and detailed terms “stroma,” “fibrosis,” and “inflammation” that specifically describe tumor-infiltrating lymphocytes and regenerating lymphatic structures, as well as confirmed their utility in standardizing the pathological assessment of the efficacy of immunotherapy (28). However, an abstract from the ASCO meeting in 2020 indicated that in 24 NSCLC patients with stage I–IIIA treated with neoadjuvant nivolumab or nivolumab + ipilimumab, the heterogeneity of CT images was significantly increased in patients who achieved MPR, possibly reflecting increased T cell infiltration or tumor necrosis (29) and suggesting that imaging features are associated with treatment MPR. Therefore, further studies are needed to determine the effectiveness and feasibility of neoadjuvant therapy for patients with early-stage lung cancer based on non-invasive markers of imaging characteristics combined with pathological markers in a larger cohort of patients. In addition, neoadjuvant immunotherapy has diverse pathological changes and is complex and cumbersome to evaluate, which requires data from multiple large randomized clinical trials and long-term follow-up to verify the reliability of MPR and irPRC as surrogate markers of RFS and OS. With this background to establish a valid surrogate endpoint, there are multiple problems to be addressed in neoadjuvant studies: (i) The lack of a uniform endpoint in ongoing studies makes the situation complicated; (ii) nearly all trials of neoadjuvant immunotherapy are multiple small non-randomized “exploratory” phase II studies; (iii) confounding regimen incorporating single immunotherapy as well as chemoimmunotherapy makes the interpretation and comparison of study results difficult; and (iv) given the large ongoing phase III adjuvant immunotherapy studies, it is challenging to enroll sufficient patients into large neoadjuvant studies.

## Predictive Biomarkers of Neoadjuvant Immunotherapy

At present, the overall efficacy of neoadjuvant immunotherapy fluctuates widely, ranging from 10 to 90%, and is limited by different therapeutic means. Therefore, it is difficult to correctly predict which populations could benefit more from neoadjuvant immunotherapy. Effectively predictive biomarkers will be specific for the selection of patients in clinical trials of ICI neoadjuvant therapy. Currently, markers that are being collected in phase III clinical trials related to the efficacy of immunotherapy include the following four major categories: (i) tumor cell-associated biomarkers, including PD-L1 expression, tumor mutation burden (TMB), DNA damage response (DDR) pathways [e.g., DNA mismatch repair deficiency (dMMR)/microsatellite instability (MSI)], specific mutant gene pathways (e.g., IFN-γ pathway, KRAS and STK11 mutation), and neoantigen load; (ii) TME-related biomarkers, including PD-L1 expression, and tumor-infiltrating immune cells, including immune cells with specific phenotypes (e.g., CD39^+^CD8^+^T, CD4^+^T cells, FOXP3^+^T cells, NKp46^+^ cells), diversity of immune repertoires [e.g., richness and clonality of tumor-infiltrating lymphocytes (TILs), T cell receptor (TCR) repertoire], and immune status score; (iii) liquid biopsy-related biomarkers, including peripheral blood cells [e.g., CD45RO^+^/CD8^+^T cells, CD4^+^ICOS (inducible T cell co-stimulator)^+^T cells, circulating tumor cells (CTCs)], circulating tumor DNA (ctDNA), and other circulating molecular biomarkers (e.g., exosomes, cytokines, and inflammatory factors); and (iv) host-related markers, involving general characteristics (e.g., gender, age, and body fat distribution), intestinal commensals, and host germline genetics [e.g., human leukocyte antigen (HLA) diversity and other specific mutations].

In early lung cancer, some widely studied markers, such as PD-L1, TMB, and immune status of the TME, were first explored preliminarily. First, as a proposed test by the U.S. FDA, PD-L1 on tumor cells is considered a biomarker of anti-PD-1 inhibitors, especially for NSCLC patients to receive pembrolizumab. Markers analysis of the NEOSTAR study (19) showed that pretreatment PD-L1 expression was higher in responders than in non-responders (80% vs. 1%, *p* = 0.015). The percentage of viable tumor cells was lower in patients with PD-L1 > 1%. However, several studies hold different views that MPR was not associated with PD-L1 expression, such as the CheckMate 159 study (11) and LCMC3 study (14), highlighting the limitations of the PD-L1 assay as an effective predictor for neoadjuvant immunotherapy. Besides, high TMB is an emerging potential predictive biomarker for MPR after adjuvant and neoadjuvant immunotherapy, meaning the total number of mutations present in tumor specimens. In the study of Forde et al. (11), anti-PD-1 therapy increased the number of neoantigen-specific T cell clones in tumor and peripheral blood in resectable NSCLC, suggesting that TMB may be used as a predictor of treatment response. Nevertheless, TMB alone is not effective in predicting treatment response/survival in patients, which needs to be further explored. The immune status of the TME also needs to be analyzed. In the LCMC3 study (14), compared with patients without MPR, patients with MPR had lower baseline levels of T cells and NK cells, but after neoadjuvant therapy, these patients experienced expansion of NK cells and granulocytes and increased abundance of dendritic cells and B cells in lymph nodes, as well as decreased abundance of monocytes, suggesting that ICIs play a key role in preoperative activation of tumor-specific immune killing. A study in patients with stage III melanoma treated with neoadjuvant ICIs found that expansion of tumor-resident T cell clones and a favorable IFN-γ gene signature were associated with RFS (30). In addition, liquid biopsy is a promising tool to non-invasively monitor response to neoadjuvant or adjuvant immunotherapy. The CheckMate 159 study explored the relationship between efficacy and specific expansion of tumor-specific T cells in peripheral blood and found that the clonal subtype of tumor-specific T cells increased continuously with treatment in patients with MPR and persistent disease-free status, but it gradually decreased in patients with non-MPR and recurrence (31). Therefore, dynamic remodeling of tumor-specific T cells in peripheral blood can serve as a predictive biomarker for neoadjuvant immunotherapy. ctDNA appears to be present in 50–95% of stage I to III patients (32, 33), suggesting that changes in ctDNA before and after neoadjuvant immunotherapy may be another more broadly applicable biomarker. The clearance of ctDNA and the expansion of tumor-specific T cells in peripheral blood may early monitor the treatment response and recurrence (34). However, whether ctDNA and tumor-specific T cells in peripheral blood are associated with MPR or even OS or DFS is not clear. The NADIM study is performing an immune repertoire profiling of peripheral blood TCR in patients with stage IIIA NSCLC receiving immunotherapy in combination with chemotherapy. In addition, the blood collection process, blood sample storage conditions, and centrifugation speed of separated plasma are all limiting factors associated with clinical practice (35, 36). To explore the clinical utility of these tests in patients receiving adjuvant and neoadjuvant immunotherapy, future trials should include serial collection of liquid biopsies.

The above results of biomarker studies in early-stage tumors are approximately similar to those in advanced tumors. However, other studies have also shown that the exploratory results in early-stage tumors are inconsistent. For example, in advanced lung cancer, driver gene mutations like EGFR and ALK have been shown to be associated with reduced response rates to ICIs and low TMB; therefore, the FDA does not recommend first-line ICI treatment in patients with EGRF or ALK-positive tumors (37, 38). But in the LCMC3 study of neoadjuvant immunotherapy, 37.5% (3/8) of EGFR/ALK-positive patients had pathological response (14), suggesting that NSCLC with specific gene mutations is not necessarily a limitation and contraindication for neoadjuvant immunotherapy in early-stage tumors. However, since the results were observed only in a small number of patients, several neoadjuvant trials have excluded EGFR/ALK-positive patients. In advanced disease, the role of ICIs in populations with driver mutations is also not clear. Therefore, further studies need to be conducted to benefit more patients.

A study developed a quantitative system pharmacology (QSP) model to predict response to neoadjuvant and adjuvant anti-tumor immunotherapy of human NSCLC (39). This model integrates knowledge of tumor growth, antigen processing and presentation, T cell activation and distribution, antibody kinetics, and immune checkpoint kinetics. The results showed that, in addition to TMB, the number of effector T cells and regulatory T cells in the tumor and blood was a predictor of responders. This suggests that it may be promising to obtain the most effectively comprehensive predictive markers by extracting features with large samples and multiple dimensions and constructing multivariate models using machine learning. Given the availability of preoperative and postoperative specimens during the study, neoadjuvant therapy has some advantages in the discovery and exploration of predictive markers. Although multiple studies have explored predictive markers of efficacy to neoadjuvant immunotherapy, the results have not been consistent, and considering that they are only preliminary exploratory analyses, the credibility of results still deserves further scrutiny. At present, there is no standard predictive marker for efficacy to neoadjuvant immunotherapy, and prospective large-scale studies are still needed to identify the most effective duration of neoadjuvant therapy and the best predictive biomarker of response.

## Conclusion and Future Prospects

In view of the high risk of postoperative recurrence of resectable NSCLC, many perioperative treatment methods are being continuously explored to prevent postoperative recurrence and obtain long-term survival benefit, such as chemotherapy, targeted therapy, and immunotherapy, continuously enriching the content of neoadjuvant and adjuvant therapy and bringing a brand-new different era for operable patients. ICIs are currently a hot topic and breakthrough point in cancer therapy and are gradually applied in earlier NSCLC. Through continuous conduction of relevant clinical trials, ICIs have made many breakthroughs with significant improvement in efficacy and in the exploration of response evaluation and predictive biomarkers. Although important clinical trials are still ongoing, exciting preliminary results have been obtained from the completed trials, in which the MPR of immune monotherapy reached 22–45%, the MPR of immunotherapy combined with chemotherapy reached 50–83%, and the safety was good, indicating that neoadjuvant immunotherapy is a promising treatment strategy for patients with resectable lung cancer. Studies currently ongoing include (i) phase III adjuvant chemoimmunotherapy studies, (ii) multiple small phase II neoadjuvant immunotherapy studies, (iii) small phase II chemoimmunotherapy studies, and (iv) phase III neoadjuvant chemotherapy plus immunotherapy followed by different lengths of postoperative adjuvant immunotherapy.

Nevertheless, neoadjuvant and adjuvant therapy with ICIs for NSCLC are still in the initial stage of exploration, and there are still many challenges for clinical adaptability and feasibility. First, as we have seen in some studies, neoadjuvant immunotherapy may affect the timing of surgery and increase the difficulty and risk of surgery due to its side effects or disease progression, suggesting that it is particularly important for screening treated patients. irAEs may still occur during and after treatment, especially in combination with dual immunotherapy. Also, the assessment of pseudoprogression and hyperprogression problems of immunotherapy still needs more exploration and evidence. Second, the study design of optimal treatment mode of neoadjuvant immunotherapy, including the choice of immunotherapeutic drugs, application cycle, and time point, is still challenging. On the one hand, most studies on neoadjuvant therapy will continue using adjuvant chemotherapy, adjuvant immunotherapy, or consolidation immunotherapy after surgery, which may affect the accurate observation of the efficacy of neoadjuvant therapy; on the other hand, most of them are preliminary exploratory studies in phases I–II, while there are few prospective phase III studies. The existing phase III studies lack a conventional chemotherapy control trial, so it is unknown whether the efficacy of neoadjuvant therapy could have a significant impact on existing study results. In addition, whether alternative endpoints (such as pCR and MPR) can predict the survival rate and have a positive impact on DFS or OS, as well as the development and validation of reliable evaluation criteria for response to neoadjuvant immunotherapy, is not clear. The treatment decision needs to be carefully selected after balancing the factors, such as treatment efficacy, safety, and surgery rate, to maximize patient outcomes. Finally, there is no standardized biomarker to identify the patient population who can benefit or develop irAEs. Although some available markers have been explored, including PD-L1, TMB, and liquid biopsies proposed recently, none have sufficient evidence to directly correlate with MPR or OS. In future studies, it is most important to develop biomarkers that reflect both tumor–immune system and immune system–host interactions based on the characteristics of immunotherapy itself to aid clinicians identify the patient population that will benefit the most from neoadjuvant immunotherapy. Of note, other treatment modalities are also being continuously explored; for example, the ADAURA study (ASCO abstract #LBA5) suggests that adjuvant chemotherapy followed by EGFR-TKIs is expected to be an effective treatment regimen. A multidisciplinary collaborative model for early-stage lung cancer is constantly being explored and developed, all of which have prompted better application of immunotherapy in the surgical treatment of resectable NSCLC, and more and larger prospective clinical studies are expected in the future to develop the best treatment strategy.

## Author Contributions

RB carried out the primary literature search, drafted and revised the manuscript, and participated in discussions. LL, XC, NC, and WS helped modify the manuscript. JC carried out the literature analysis, drafted and revised the manuscript, and participated in discussions. All authors read and approved the final manuscript.

## Conflict of Interest

The authors declare that the research was conducted in the absence of any commercial or financial relationships that could be construed as a potential conflict of interest.

## Abbreviations

ACSO: American Society of Clinical Oncology
ALK: anaplastic lymphoma kinase
CTCs: circulating tumor cells
ctDNA: circulating tumor DNA
DC: dendritic cell
DDR: DNA damage response
DDR: DNA damage response
DFS: disease-free survival
dMMR: DNA mismatch repair deficiency
EFS: event-free survival
EGFR: epidermal growth factor receptor
FDA: Food and Drug Administration
HLA: human leukocyte antigen
ICIs: immune checkpoint inhibitors
ICOS: inducible T cell co-stimulator
irAEs: immune-related adverse events
irPRC: immune-related pathological response criterion
MPR: major pathologic response
MSI: microsatellite instability
NCCN: National Comprehensive Cancer Network
NK cells: natural killer cells
NSCLC: non-small cell lung cancer
ORR: objective response rate
OS: overall survival
pCR: pathologic complete response
PD-1: programmed cell death-1
PD-L1: programmed cell death-ligand 1
PFS: progression-free survival
PR: partial response
PsPD: Pseudoprogression
QSP: quantitative system pharmacology
RATS: robot-assisted thoracic surgery
RFS: relapse-free survival
SD: stable disease
SLD: sum of lesion diameters
TCR: T cell receptor
TILs: tumor-infiltrating lymphocytes
TIM: tumor-infiltrating myeloid cells
TMB: tumor mutation burden
TME: tumor microenvironment
TMU: tumor metabolism uptake
TRAEs: treatment-related adverse events
Treg: T regular cell
VATS: video-assisted thoracic surgery.
